# Social Media Reactions to Sex Toy Criminalization in Ghana: Cross-Sectional Sentiment and Text Network Analysis

**DOI:** 10.2196/91422

**Published:** 2026-08-11

**Authors:** Anthony Senanu Agbeve, Celestin Niyomugabo, Celestin Hakizimana, Daniel Yaw Fiaveh, Amanda Yad-El Ugboji, Caroline Pukall, Martina Anto-Ocrah

**Affiliations:** 1 Department of Sociology and Anthropology, University of Cape Coast, Faculty of Social Sciences, Arku Korsah Road, Cape Coast, Central, CC-192-1692, Ghana, 233 240217647; 2 Vonsung, Kigali, Rwanda; 3 Department of Medicine, Division of General Internal Medicine, University of Pittsburgh, Pittsburgh, PA, United States; 4 Department of Psychology, Queen's University, Kingston, ON, Canada

**Keywords:** sexuality, sexual pleasure, sex toy, LGBTQ+, Ghana, lesbian, gay, bisexual, transgender, and queer

## Abstract

**Background:**

Sexuality discourse in Ghana has become more divided, especially around gender and lesbian, gay, bisexual, transgender, and queer rights, leading to new antigay laws criminalizing lesbian, gay, bisexual, transgender, and queer activities, and other forms of sexual practices. One of the most controversial pieces of legislation, previously titled “The Promotion of Proper Human Sexual Rights and Family Values Bill,” proposed in 2021, sought to criminalize the use of sex toys, sparking opposition from leading political figures. The then minister of communication, Ursula Owusu-Ekuful, a staunch women’s rights advocate, publicly opposed the clause criminalizing sex toys, stating that such initiatives infringed on women’s sexual autonomy. Her stance sparked heated debates on several social media platforms, reflecting the broader tensions surrounding conversations about sexuality in Ghana. To better understand the public’s reactions to the minister’s counterproposal, we analyzed comments from various social media platforms.

**Objective:**

Specifically, we (1) investigated public perceptions and sentiments regarding the criminalization of sex toys as expressed on social media, and (2) identified whether sentiments differed across social media platforms (eg, are comments on X [X Corp] more negative compared to Instagram or Facebook [Meta Platforms, Inc]?).

**Methods:**

Using Python (Python Software Foundation) as a web scraping tool, we extracted public comments from Instagram, Facebook, X, and YouTube (YouTube, LLC). Comments were analyzed using natural language processing to categorize sentiments (positive, negative, and neutral, indicating support for opposition to the bill, support for introducing the bill, and no clear stance in either direction, respectively), and text network analysis was used to identify keywords and their relationships to central themes. We used conditional probability analyses to determine the likelihood that a given comment was classified as negative, positive, or neutral, depending on its source (Instagram, X, Facebook, or YouTube).

**Results:**

A total of 951 comments were retrieved: 62.7% (n=596) from Instagram, 28.2% (n=268) from X, 6.2% (n=59) from Facebook, and 2.9% (n=28) from YouTube. Negative sentiments dominated (n=631, 66.4%), with only 13.3% (n=126) positive and 20.4% (n=196) neutral sentiments. Strongly disapproving language—some directed towards the minister—was common. This included terms such as “lesbian,” “disgrace,” and “disgusting.” X showed the highest proportion of negative sentiment (190/268, 70.9%), followed by Facebook (41/59, 69.5%), YouTube (18/28, 64.3%), and Instagram (382/596, 64.1%). Conditional probability analysis showed that Facebook and X had the highest likelihood of negative comments (0.7), and sentiment distribution differed significantly across platforms (*P*<.001). Text network analysis revealed recurring themes of morality, politics, stigma, and gender.

**Conclusions:**

Our findings highlight deep-rooted cultural resistance to non-heteronormative sexual behaviors in Ghana and also suggest that women’s advocacy for sexual autonomy may elicit patriarchal backlash. However, emerging positive voices suggest gradual shifts toward recognizing sexual pleasure as a right, pointing to the need for comprehensive sexuality education that emphasizes positive sexuality and the multifaceted nature of the human sexual experience.

## Introduction

The Ghanaian understanding of sex and sexuality is deeply embedded in cultural and religious doctrines that shape societal perceptions, norms, and expectations about sex and reproduction [1,2]. Ghanaian culture largely emphasizes heterosexual relations as the foundation of kinship and lineage continuity. Christianity, Islam, and Ghanaian spirituality (also known as Ghanaian traditional belief systems), the dominant religions or spiritualities in Ghanaian kinship, uphold conservative views on sex and sexuality, often condemning publicly nonheteronormative sex and sexualities (largely because of the cultural values of reproduction as a product of sex) and reinforcing “traditional” gender roles of male dominance and the important role of the penis [3,4]. Clergy and leaders from religious faiths and social institutions (schools, markets, workplaces, and other public spaces) work together in endorsing the moral codes of sex through their teachings and practices, including influencing not only personal beliefs but also courting politicians [5] and their political parties as well as national debates and policies on sexual and reproductive rights [6,7].

Ghana’s lesbian, gay, bisexual, transgender, and queer (LGBTQ) community has gained visibility by aligning with global movements for LGBTQ rights, such as Rightify Ghana, LGBT Rights Ghana, the OHF initiative, and Key Watch Ghana, which lobby through social groups and research and occasionally engage with the media [8]. However, the growing visibility of these LGBTQ groups and their advocacy for sexual rights and freedom has ignited public backlash from diverse stakeholders, including traditional leaders, parents, the clergy, academics, human rights advocates, the media, and religious groups, including the Christian Council of Ghana, the Ghana Catholic Bishops Conference, and the Coalition of Muslim Organizations Ghana [9,10]. Ghana’s Supreme Court has also emerged as a key actor in shaping legal jurisprudence on sexual practices and LGBTQ-related matters. In its July 24, 2024, ruling on the constitutionality of Section 104(1)(b) of the Criminal Offenses Act (Act 29), the Supreme Court reaffirmed that “unnatural” carnal knowledge, and the use of sex toys such as dildos and vibrators, was unlawful [11]. This shows that the contestation over LGBTQ rights and sexual practices in Ghana extends beyond activism into legal, cultural, and religious arenas, setting the stage for deeper debates on sexuality-related issues in the country.

On August 2, 2021, a bill titled “The Promotion of Proper Human Sexual Rights and Ghanaian Family Values Bill” was laid before Ghana’s Parliament. The purpose of the bill was to expand and strengthen legal prohibitions on LGBTQ-related activities, including advocacy, promoting or funding LGBTQ activities, and public display of same-sex-related activities. Even though the bill was passed by the Ghanaian parliament under the revised title “The Human Sexual Rights and Family Values Bill, 2025,” amid stiff opposition from feminist rights activists in and out of the Ghanaian parliament, the then president, Nana Addo Dankwa Akufo-Addo, did not assent to the bill. There were several oppositions even within the then ruling party (The New Patriotic Party) and the opposition party (The National Democratic Congress) to aspects of the bill, which some described as draconian. One member of the opposition, prior to its passage, Madam Ursula Owusu-Ekuful, former Minister of Communication and also a known women’s rights activist, raised concerns about the criminalization of sex toys included in the bill. According to her, the proposed Clause 3(c) of the bill, which seeks to criminalize sex with an inanimate object, infringes on the sexual rights of many Ghanaians, particularly women and heterosexual couples, who may use sex toys, vibrators, and other sexual aids to enhance their sexual pleasure [12]. Owusu-Ekuful called for the deletion of clause 3(c) of the proposed bill, stating that:

This may create unintended consequences because sexual intercourse between a man and an inanimate object or between a woman and an inanimate object could necessarily include sexual intercourse with all manner of aids, including sex toys that couples use to enhance their sexual experience.

Public reactions to her comments reflected a deep societal divide. While some applauded her audacity in openly discussing a topic often considered taboo, others criticized her position and accused her of endorsing practices perceived as inconsistent with Ghanaian cultural and religious values. Given that her comments were made within debates surrounding the anti-LGBTQ bill, they could be interpreted in multiple ways. Some sections of the public may have viewed her remarks as a defense of sexual autonomy more broadly, including the use of sex toys by heterosexual couples, adults, and individuals engaged in nonheteronormative sexual relationships and practices. Others may have understood them through prevailing anxieties about LGBTQ rights and broader public narratives that portray Western governments and international actors as exerting pressure on Ghana to recognize the rights and freedoms of sexual minorities. However, the controversy also became distinctly gendered because the comments were made by a prominent female politician and women’s rights advocate who has long been accused of being a member of the queer community. Consequently, many public responses focused on questions of women’s sexual agency, pleasure, and bodily autonomy, issues that remain highly contested within Ghanaian society.

The reactions to Owusu-Ekuful’s comments also highlight the growing role of social media in shaping and contesting public discourses on sexuality-related issues, including sexual rights and bodily autonomy. Social media has transformed narratives around sex and sexual pleasure seeking with sex toys and other pleasure aids, with platforms such as Instagram, Facebook (Meta Platforms, Inc), and TikTok (ByteDance Ltd) increasingly serving as spaces for promoting and fostering open discussions about sexual autonomy and pleasure seeking [8,13-15]. The growing visibility of these platforms, alongside the expansion of the sex market to online spaces, signals a significant cultural shift as individuals are increasingly finding alternative avenues to discussing and learning about sex. This development reflects the gradual normalization of conversations around sexual autonomy, beyond traditional heteronormative expectations. Scholarship on sexuality has long highlighted how sexual practices and identities are shaped through social norms, power relations, and mechanisms of regulation [16-18]. Within African contexts, however, scholars (such as Stella Nyanzi, Sylvia Tamale, and Akosua Adomako Ampofo) have drawn attention to the specific ways gender, sexuality, and pleasure are negotiated within local cultural and political realities. The shifting landscape of discourse and resistance observed in digital spaces aligns with these African feminist perspectives, which view women not as passive recipients of cultural norms but as active agents capable of making self-reflective choices about their bodies and desires, even within highly regulated environments [19,20]. This perspective is particularly relevant in conservative heteropatriarchal contexts, where women’s expressions of sexual autonomy are frequently contested and subjected to social regulation. Although the National Democratic Congress government led by President John Dramani Mahama has yet to assent to the bill, which has been reintroduced in the Ninth Parliament of the Fourth Republic, it remains unclear whether the concerns previously raised by Ursula Owusu-Ekuful have been adequately addressed. Both the president and the speaker of parliament, Alban Kingsford Sumana Bagbin, have called for broader consultations and a reconsideration of the bill. From this perspective, we argue that the concerns raised by Ursula Owusu-Ekuful resonate with broader feminist critiques and sections of Ghanaian public opinion that question state regulation and surveillance of sexuality and reproductive rights. These concerns underscore the importance of wider public engagement and continued debate on issues of autonomy, bodily integrity, and the limits of state intervention in intimate life.

Therefore, using publicly available data, this study analyzed social media comments regarding Ursula Owusu-Ekuful’s stance on the criminalization of sex toys. We recognize that debates surrounding sex toys in Ghana may evoke broader concerns relating to sexuality, LGBTQ rights, sexual autonomy, and the legitimacy of nonreproductive sexual practices. Accordingly, public responses to Owusu-Ekuful’s comments may reflect attitudes towards a range of sexual identities and practices. Nevertheless, we focus particular attention on women because the public controversy has become strongly gendered, with reactions frequently centering on the appropriateness of women discussing, seeking, or exercising sexual pleasure. Against this backdrop, our two aims were to (1) investigate public perceptions and sentiments regarding the criminalization of sex toys as expressed on social media, and (2) identify whether sentiments differed across social media platforms (eg, X [X Corp], Instagram, and Facebook). Our overarching goal was to explore the narratives surrounding sexuality and bodily autonomy and to examine how these discourses shape sexual agency in contemporary Ghana, particularly for women.

## Methods

### Research Design

This study uses a computational content analysis design to examine publicly available social media discourse on sex toy legislation in Ghana. The dataset consists entirely of textual data drawn from user comments posted on Instagram, X, Facebook, and YouTube. To analyze these comments, we combined natural language processing techniques with qualitative interpretation. Computational methods were used to identify sentiment patterns, keyword associations, and variations across social media platforms, generating outputs such as sentiment frequencies, conditional probabilities, and *χ*² statistics. These measures were used to describe and compare patterns within the data rather than to establish causal relationships [21,22]. The interpretation of results remained grounded in the content, context, and meanings expressed in the comments. This approach aligns with emerging traditions in computational social science that combine machine learning and natural language processing techniques with qualitative modes of inquiry to analyze large volumes of textual data.

### Data Collection

Data was collected from the following social media platforms: Instagram, X, Facebook, and YouTube, capturing online engagements between December 17-31, 2023, using Python (Python Software Foundation), a programming language for data analysis, as a web scraping tool. Publicly available information was extracted from these platforms that focused on the minister’s speech regarding the criminalization of sex toys in Ghana. Data cleaning was performed on all scraped data and involved removing duplicates, empty comments, and stop words (such as oh, hello, or, and is). Natural language processing methods, specifically lemmatization, were used to reduce words in the text to their root forms. Lemmatization served a critical function by standardizing texts, improving text categorization precision, and aiding feature extraction and semantic analysis [23]. We also removed emojis from the dataset during the data-cleaning phase to prevent misclassification of sentiment scores. For example, comments with laughing emojis might be classified as positive when they are actually attached to negative or mocking comments.

### Data Analysis

The data were analyzed using a three-step process:

1. The first step was sentiment analysis. Sentiment analysis was used to identify the emotional tone underlying comments on these social media platforms. This helped us organize and categorize public reactions to Ghana’s sex toy legislation. We used a pipeline sentiment analysis method, which sequentially preprocesses the text, extracts key linguistic features, and applies machine-learning models to classify each comment. Drawing on data mining, machine learning, AI, and computational linguistics techniques [24], this approach enabled us to determine whether sentiments were positive, negative, or neutral. Positive sentiments reflected support for opposition to the bill, negative sentiments indicated support for introducing the bill, and neutral sentiments showed no clear stance in either direction.
2. Second, text network analysis was used to visualize the structure and relationships of emerging keywords based on their usage and co-occurrence. In the visualization, each keyword is represented by a node, and lines (edges) are used to connect nodes that co-occur in similar contexts. The size of each node indicates how frequently the corresponding word appears in comments on all platforms. Larger nodes represent words that appear more frequently, indicating they are central themes in the comments. The visualizations generated by the text network analyses reveal clusters, or “communities,” of words, where nodes within the same community are more densely connected but have fewer connections to nodes in other groups.
3. Lastly, we conducted a comparative analysis of the comments to better understand which social media platforms had the most polarizing sentiments. This involved examining patterns and differences in reactions across the various social media platforms to identify platform-specific trends. Conditional probability analyses were conducted to determine the likelihood that a given comment was classified as negative, positive, or neutral based on its source being Instagram, X, Facebook, or YouTube. We used the following formulas where:
  - *P*(*AorB*) is the conditional probability of event A given that event B has occurred.
  - *P*(*A*∩*B*) is the joint probability of events A and B occurring simultaneously.
  - *P*(*B*) is the probability of event B occurring.

### Ethical Considerations

This study involved the passive analysis of publicly available social media comments and did not require direct interaction with users. Consequently, ethical approval and informed consent were not required [25]. To protect users’ privacy and minimize the risk of harm, usernames and links to the original posts were not reported, and quotations were presented without identifying information [26]. The study adhered to the ethical guidelines of the Association of Internet Researchers for research involving internet-based data [27].

## Results

### Overview

In total, 951 comments were scraped from four data sources (ie, Instagram, X, Facebook, and YouTube) after data cleaning. The majority of the comments, 62.7% (n=596), came from Instagram, while 28.2% (n=268) came from X, 6.2% (n=59) from Facebook, and 2.9% (n=28) from YouTube.

### Aim 1: Public Perceptions and Sentiments Regarding the Criminalization of Sex Toys

We analyzed social media comments to understand how members of the public responded to debates surrounding the proposed criminalization of sex toys. Specifically, we examined the distribution of positive, negative, and neutral sentiments expressed in relation to Ursula Owusu-Ekuful’s comments and the broader issue of sex toy use. Representative excerpts are presented to illustrate the views, concerns, and positions reflected within each sentiment category.

**Figure 1.**
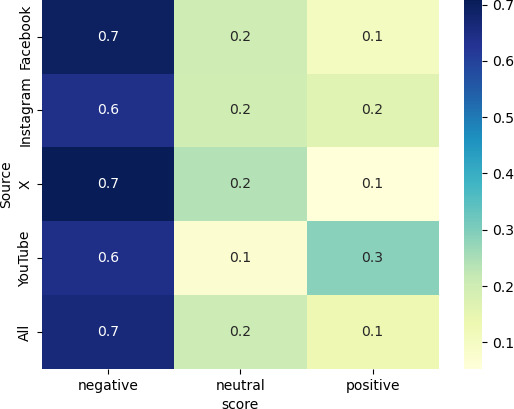
Probability distribution of scores by source.

Out of the 951 comments, the pipeline sentiment analysis classified 631 (66.4%) as negative sentiments, 126 (13.3%) were positive, and the remaining 194 comments (20.4%) were classified as neutral (Figure 1). Negative sentiments included keywords like “stupid,” “useless,” “foolish,” “disgrace,” and “disgusting.” Phrases such as “She was a lesbian all through secondary school. I am not surprised. That’s why she doesn’t respect men…,” “She is a disgrace to the Ghanaian culture,” “this is exactly why our culture is losing its values. We should not be encouraging such things,” and “people in leadership should promote morals, not defend things that bring shame to society” underscored the discontent and opposition to the minister’s stance (Table 1).

Positive sentiments included words like “love,” “joy,” “better,” and “enjoy.” This was emphasized with phrases such as “Sex toys do not kill... they always bring joy to women who do not have boyfriends.” and “She makes a very valid point. Heterosexual couples often employ aids to enhance their sexual experience; therefore, blanket criminalization of sex aids is rather misguided…,” “adults should be free to make their own choices as long as nobody is harmed,” “there is nothing wrong with couples using aids to improve their relationship,” and “I support her on this because sex toys are for general use and not limited to only gays” (Table 1).

**Table 1.** Sentiments and keywords.

Sentiment category	Comments	Keywords
Negative	“I suspect this woman is going to be freaky in the bedroom a lot.” “She was a lesbian all through secondary school. I am not surprised. That’s why she doesn’t respect men…” “If you give positions to gays and lesbians, these are the ideas they bring on board.” “Men can do the work, but she is here talking about sex toys. She is a disgrace to the Ghanaian culture.” “People in leadership should promote morals, not defend things that bring shame to society.” “This is exactly why our culture is losing its values. We should not be encouraging such things.” “People in leadership should promote morals, not defend things that bring shame to society.”	Freaky, gay, lesbian, suspect, culture, respect, disgrace, morals, disgusting, and values
Neutral	“So far as it doesn’t harm anyone, why the need for such laws?” “There are more pressing issues in this country than debating sex toys.” “It’s better to focus on improving healthcare than criminalizing personal choices.” “Let’s have a meaningful discussion about priorities in this country.” “Whether people use sex toys or not is their personal business.” “I do not see why Parliament should spend so much time debating this issue.” “Everyone is entitled to an opinion, but there are bigger challenges facing the country.”	Harm, pressing issues, personal choices, personal business, law, and priorities
Positive	“Sex toys do not kill... they always bring joy to women who do not have boyfriends.” “She has every right to use sex toys. Leave her alone…” “She makes a very valid point. Heterosexual couples often employ aids to enhance their sexual experience; therefore, blanket criminalization of sex aids is rather misguided…” “I support her on this because sex toys are for general use and not limited to only gays.” “Adults should be free to make their own choices as long as nobody is harmed.” “There is nothing wrong with couples using aids to improve their relationship.” “She is right. The government should focus on protecting rights rather than policing private lives.”	Joy, sex toys, rights, support, autonomy, and freedom

Neutral comments reflected a segment of the population whose opinions were either ambivalent or not clearly articulated. This included phrases such as “So far as it doesn’t harm anyone, why the need for such laws?”, “There are more pressing issues in this country than debating sex toys,” “I do not see why Parliament should spend so much time debating this issue,” “Everyone is entitled to an opinion, but there are bigger challenges facing the country,” and “It’s better to focus on improving health care than criminalizing personal choices” (Table 1).

Overall, the sentiment analysis suggests that public reactions to Ursula Owusu-Ekuful’s comments were predominantly negative, that is, they indicated support for the proposed criminalization of sex toys. While a smaller proportion of comments expressed support for her position or remained neutral, opposition to her stance was the dominant sentiment across the comments analyzed.

### Text Network Analysis of Keywords

**Figure 2.**
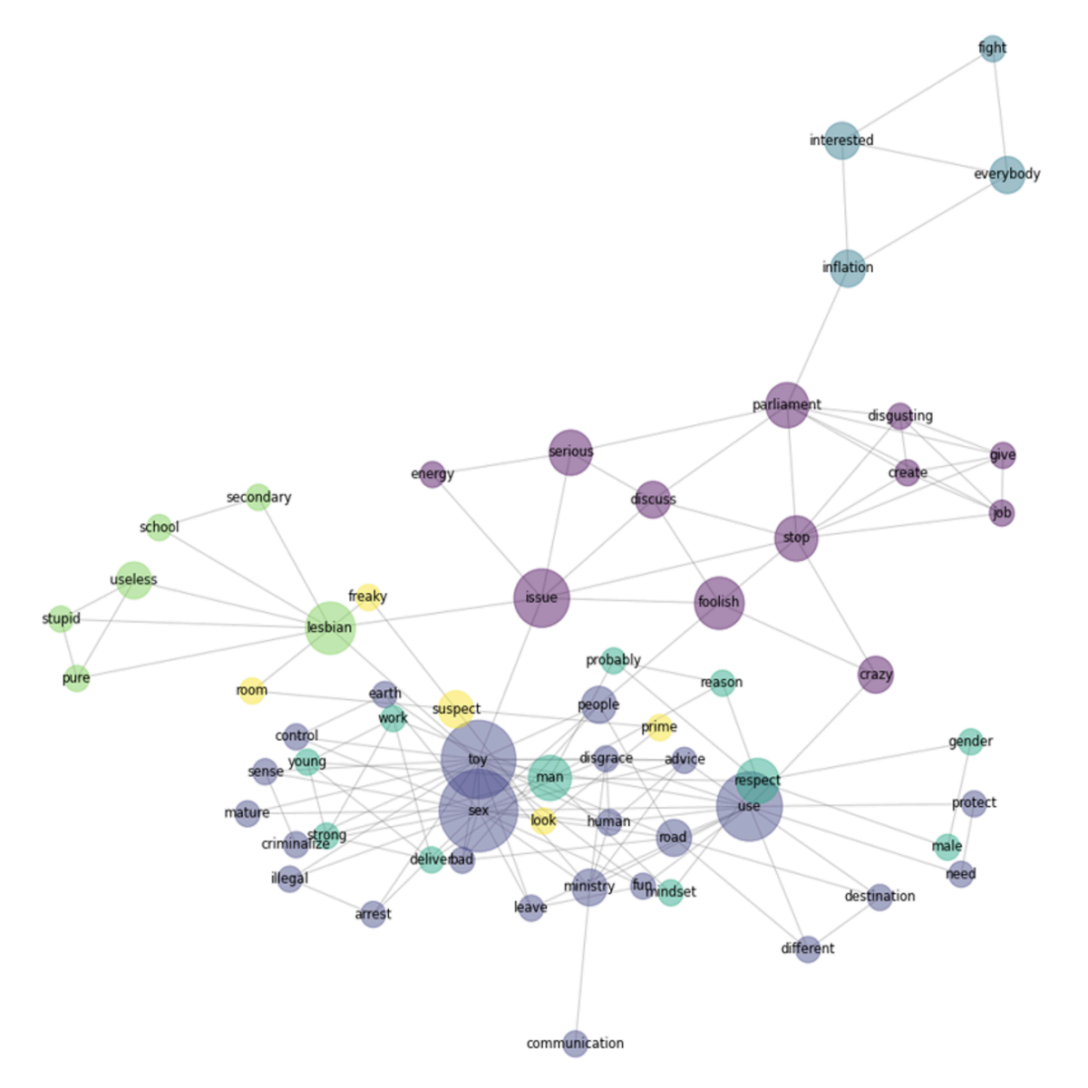
Text analysis of keywords based on community detection algorithms.

To complement the sentiment analysis, we conducted a text network analysis of keywords to identify dominant patterns of word co-occurrence and association within the social media discussions. This approach provides insight into how words co-occurred in the comments and highlights the different strands of discourse that shaped public reactions to the proposed criminalization of sex toys and the Minister’s comments. In Figure 2, the results of the text network analyses are shown. Node sizes are determined based on the number of comments each word appears in. Larger nodes (words) indicate higher frequency or occurrence in the comments. These larger nodes may represent more significant topics or commonly discussed issues. The nodes were color-coded to differentiate the various communities identified in the analyses. For example, the purple community centers on words such as “issue,” “foolish,” “disgusting,” “energy,” and “jobs.” These sentiments suggest that some individuals are unbothered by what people do in the private spaces of their bedrooms, as they do not consider sexuality-related issues a priority that warrants state intervention. The majority in this community are frustrated by what they perceive to be misplaced priorities by the government.

The mint green community features words such as “lesbian,” “useless,” and “stupid.” This cluster mostly reflected negative attitudes towards the minister’s statements. The sentiments expressed within this cluster suggest resistance to sexual diversity and opposition to the minister’s position. The dark blue community, characterized by words such as “control,” “illegal,” “criminalize,” and “disgrace,” points to discussions focused on law, authority, and morality. Sentiments in this community frame the discussion in terms of legality and morality, asserting that actions should be taken against sexual practices they see as incompatible with religious and cultural values. Unlike the previous clusters, the light green community connects words such as “respect,” “mindset,” and “gender,” suggesting discussions on gender expectations and the importance of acknowledging and respecting sexual diversity.

Finally, the cyan community consists of words such as “inflation,” “everybody,” “fight,” and “interested,” which appear more isolated. Although this cluster does not directly engage in debates surrounding sexuality and governance, it suggests a broader concern for economic struggles and national priorities. The sentiments expressed by this group emphasize that economic hardship is a more pressing issue than the sexual affairs of Ghanaians. They are similar to the purple community in their concern about the government’s misplaced priorities.

Overall, the text network analysis reveals that discussions surrounding sex toys and their proposed criminalization were shaped by multiple, interconnected concerns. While some communities expressed opposition rooted in moral, legal, and anti-LGBTQ sentiments, others emphasized sexual autonomy, gender equality, and respect for individual choice. Other discussions shifted attention away from sexuality altogether, highlighting economic hardship and questioning the prioritization of sexuality-related issues within the broader national context.

### Aim 2: Differences in Sentiments Expressed Across Social Media Platforms

Sentiments expressed towards the proposed criminalization of sex toys varied across social media platforms. To examine these differences, conditional probability analysis and *χ*² tests were used to compare the distribution of positive, negative, and neutral sentiments across comments posted on X, Facebook, YouTube, and Instagram.

Conditional probability analysis showed that comments from Facebook and X had the highest likelihood of being negative (conditional probability of 0.7), followed by Instagram and YouTube (conditional probability of 0.6). Conversely, the most positive comments were posted on YouTube (conditional probability of 0.3), followed by Instagram (conditional probability of 0.2), while Facebook and X each had a conditional probability of 0.1. Neutral comments had a conditional probability of 0.2 for Facebook, Instagram, and X, compared with 0.1 for YouTube. The *χ*² test showed a statistically significant association (*P*<.001) between sentiment category (neutral, positive, or negative) and social media platform (Instagram, X, Facebook, or YouTube), indicating that the observed differences in sentiment distribution across platforms were unlikely to have occurred by chance.

The results show significant differences in sentiment across social media platforms. Comments posted on X and Facebook were more likely to express negative sentiments, while YouTube and Instagram contained relatively higher proportions of positive comments. These differences indicate that the distribution of sentiments varied by platform.

## Discussion

### Principal Findings

The study analyzed social media data to examine public sentiments surrounding state regulation and surveillance of sexuality in Ghana, with particular attention to the proposed criminalization of sex toys under the Anti-LGBTQ Bill. Drawing on public reactions to statements made by former minister of communications and member of parliament, Ursula Owusu-Ekuful, the study explored how social media users responded to provisions perceived as extending state control over intimate practices and sexual autonomy. In doing so, it examined the ways in which debates over sex toys became intertwined with broader concerns about bodily autonomy, privacy, and the governance of sexuality in contemporary Ghana.

The minister’s vocal stance drew attention to the widespread policing and stigmatization of sexual agency and the broader pattern of how sexuality issues are tightly regulated in public discourse, particularly for Ghanaian women. From a Foucauldian perspective, these forms of regulation extend beyond formal laws or state control to the ways cultural, religious, and moral norms shape how women govern their own sexual conduct. While some women may be silenced or punished for asserting their sexual autonomy, others (such as Madam Ursula Owusu-Ekuful, who is highly educated and has legal training) may find spaces to navigate and even resist these restrictions because of their social positioning. In fact, studies show that Ghanaian women are increasingly turning to sex toys in response to sexual dissatisfaction, their partners’ sexual weakness, infrequent sex, or a personal desire for additional pleasure [28]. For these women, owning and using a sex toy may be a form of sexual agency and a way of reclaiming bodily autonomy. This gendered resistance challenges the notion that sexual pleasure and fulfillment can be attained solely through penetrative sex. For married women, however, the opposite is observed. According to Fiaveh, married women are reserved about using sexual aids, even in the absence of a sexual partner, because they believe that such practices violate religious and cultural expectations [28]. This reflects how Foucault’s idea of governmentality [29] operates through the internalization of dominant moral norms, leading individuals to regulate their own behavior in ways that align with socially accepted ideals of sexuality. To them, heterosexual, penetrative sex is the natural and morally sanctioned form of sexual expression. Thus, different women in Ghana face different kinds of vulnerabilities but also find different pathways through which they may explore and exercise agency in the face of societal constraints to assert and fulfill their sexual needs.

Our findings revealed predominantly negative attitudes and sentiments toward public discussions of sexuality, particularly regarding the use of sex toys and other pleasure-enhancing products on all social media platforms. The negative sentiments expressed were not merely expressions of personal discomfort but were deeply embedded in broader societal anxieties about shifting gender and sexual norms. Much of the backlash stems from growing fears over the visibility and activism of queer and minority groups, whose demands for recognition and rights are perceived by many as a threat to the moral, religious, and cultural fabric of Ghanaian society [30,31]. However, beyond the general resistance to nonnormative sexualities, many of the negative sentiments revealed sexist undertones, targeting women who expressed or supported sexual autonomy. Women were often labeled as immoral, deviant, or “loose” simply for acknowledging or advocating for the right to sexual agency, whether through the use of sex toys or open discussion of desire and pleasure. Such views reflect a deep-seated discomfort with women asserting control over their sexual lives and reinforce the societal expectation that respectable womanhood is contingent upon sexual restraint, modesty, and subservience to male pleasure [28,32,33]. These notions highlight the ongoing policing of female sexuality and how patriarchal norms attempt to suppress women’s sexual agency. It also shows how negative public reactions to women’s sexual autonomy are not merely cultural expressions but active mechanisms of control that seek to limit women’s bodily autonomy and right to pleasure.

The data also suggest that discussions about sex toys do not merely challenge acceptable forms of sexuality but also provoke discomfort because of their perceived association with same-sex sexual expression. In this context, sex toys were not viewed as legitimate pleasure-enhancing tools but as symbolic threats to the socially and religiously prescribed heteronormative order. Their connection to alternative sexual practices, especially those that center on female pleasure or queer sexualities, elicits strong resistance rooted in entrenched gender norms, conservative religious teachings, and moral expectations. Sexual expressions outside the framework of reproduction and male-dominated intimacy are often framed as immoral or unnatural. This reflects broader cultural discomfort with sexual diversity and sexual autonomy. Furthermore, the findings highlight the overconcentration of normativity, a phenomenon that feminist scholar Stella Nyanzi sharply critiques. Nyanzi argues that this obsession with what is deemed “normal” constrains public imagination and actively limits the recognition and legitimacy of other diverse forms of sexual expression [19]. In Ghanaian society, early socialization and dominant religious and cultural ideologies rigidly define what constitutes “normal” or “legitimate” sexuality. Society swiftly condemns deviations from dominant sexual norms, particularly sexualities that fall outside what is considered “normal” or “legitimate” [34,35]. At the same time, a culture of silence around sexuality limits opportunities for open discussion of sexual issues and diverse forms of sexual expression [36]. This normative framework creates significant barriers to open and inclusive conversations about sexuality in the public sphere [1]. Such cultural and moral policing reinforces the status quo, marginalizes those who do not conform, and curtails the possibilities for progressive discourse on sexual rights and autonomy.

The negative reactions to the minister’s speech also underscore how women’s sexual needs are routinely suppressed within patriarchal societies and deny them a voice and agency in sexual decision-making. The backlash, particularly the use of derogatory labels such as “freaky,” “disgrace,” and “lesbian,” reveals not only discomfort with women asserting their sexual autonomy but also the societal mechanisms used to discipline and silence such vocal or assertive individuals. Women who openly discuss or advocate for sexual rights are often perceived as a threat to the masculine order and normative gender expectations, which dictate that women should be sexually passive, modest, and subordinate. Such labeling delegitimizes their sexual preferences and expressions and exposes them to heightened risks of verbal abuse, social ostracism, and physical violence. These derogatory terms and labels become ideological tools of control and deterrence, aimed at delegitimizing women’s voices and reinforcing the idea that sexual autonomy or agency for women must be controlled. Nevertheless, in private spaces, some studies in Ghana have shown that heterosexual couples reportedly engage in diverse sexual practices, including the use of sexual aids such as sex toys to enhance their sexual experiences [28,32,33,37]. This underscores the double standards that characterize Ghanaian society, where public discourse fiercely defends cultural and moral norms by shaming women and others who advocate for sexual autonomy, while diverse sexual practices continue to exist, often away from public scrutiny. Despite widespread public opposition to LGBTQ issues and other nonheteronormative sexualities, such practices have long existed within African societies [38]. Yet, open discussion of these realities remains highly constrained. This disconnect reveals that, although Ghanaians may publicly express resentment toward nonnormative sexualities and pleasure-seeking practices, such behaviors are not absent in private life. However, the public backlash and associated moral condemnation may push women and other marginalized individuals into silence. This stance may stifle broader conversations about sexual rights and autonomy. The implications are profound, not just for women’s sexual autonomy but also for their ability to define and experience pleasure on their terms.

In addition, comments such as “men can do the work” or claims that women who use sex toys “have no respect for men” reflect deeply entrenched cultural beliefs that prioritize male sexual performance and pleasure while relegating women’s sexual needs as secondary or unnecessary [32]. These perspectives are indicative of a patriarchal, heteronormative sexual framework in which women’s sexual needs are regarded as optional or contingent upon male involvement. This framing of female sexuality raises critical questions: are women only expected to participate in sex for reproduction or to satisfy men? Is sexual pleasure viewed as primarily for men? These presumptions contribute to a socio-cultural environment that actively denies women the right to pursue their sexual inclinations and make decisions about their bodies. The consequences will be far-reaching as women’s sexual autonomy is systematically restricted, and their agency is undermined when male pleasure is normalized as primary and female pleasure is devalued or stigmatized. This cultural logic not only restricts women’s capacity to explore their sexual preferences but also undermines the legitimacy of women’s sexual rights in a broader context. The normalization of male-centered sexual authority perpetuates power imbalances, which may even create environments that are conducive to gender-based violence or coercion. By framing sex toys as a rejection of male authority rather than as tools for individual or shared pleasure, such discourse reinforces the idea that women’s sexuality must remain tied to male control. Ultimately, these dynamics reproduce a social order in which women’s sexual needs are not only subordinated but also surveilled, restricted, and silenced.

Despite the associated negativities regarding female sexual expressions and autonomy, we identified some positive comments that countered the overly negative sentiments expressed about the sex toy discourse. The recognition that sex toys are intended to improve and enhance the sexual experiences of individuals, including those in heterosexual relationships, points to a gradual departure from the conventional normative ideals. For instance, comments such as “…heterosexual couples often employ aids to enhance their sexual experience; therefore, blanket criminalization of sex aids is rather misguided…*”* illustrate how heteronormative sexual norms are being contested. Narratives of this nature challenge conservative ideologies that seek to confine sexual expressions within the normative framework of penovaginal sex. This positive outlook signals a gradual shift away from the rigid structures that have long marginalized female sexual agency. In a cultural context where patriarchal and religious norms tightly regulate sexuality, there are dissenting voices advocating autonomy and the right to make self-reflective choices. This suggests that with time and continued advocacy, women and other marginalized groups will be empowered to resist restrictive cultural norms and narratives shaping sexuality discourses. This could, to a large extent, alter the cultural landscape and public perceptions of sexual rights and autonomy in Ghana.

### Strengths and Limitations

To the best of our knowledge, this is the first and only study to analyze social media comments on sex toy legislation in Ghana and to do so using AI. Our study integrates insights from media studies, law, gender and sexuality studies, and computational social science to offer a thorough analysis of public discourse on this emerging issue. In doing so, we not only pioneer a forward-looking methodology that anticipates how intelligent technologies can be used to better understand social media debates in Ghana and beyond, but we also break new ground in the intersection of legal media analysis and AI. This study has several limitations. Although the analysis focuses on public discourses surrounding women’s sexual autonomy and pleasure, the gender identities of social media commenters could not be verified. Consequently, interpretations are based on the content and context of comments, as well as the gendered debates prompted by Ursula Owusu-Ekuful’s remarks. Furthermore, because the commenter’s gender could not be established, views interpreted as supporting or opposing women’s sexual autonomy may have been expressed by men rather than women. The findings should therefore be understood as reflecting broader public discourses on women’s sexuality rather than the perspectives of women alone. This study also excludes the perspectives of offline, nonsocial-media users, potentially overlooking significant viewpoints held by less-connected or rural populations. Prior research has shown that online public discourse can diverge substantially from offline sentiment due to representativeness biases and echo chamber effects, in which people are repeatedly exposed to information and opinions that closely align with their views, reinforcing those beliefs and shielding them from opposing perspectives. This often happens in algorithm-driven platforms that prioritize familiar content [39]. In order to facilitate a more holistic understanding of the Ghanaian public opinion on sex toy legislation, future studies should incorporate offline surveys or community interviews to compare offline views with online sentiment.

### Conclusion

This study reveals entrenched societal discomfort and cultural unease over nonheteronormative sexualities and expressions in Ghana. We found that cultural and religious norms largely shaped public discussions, especially those on social media. The opposition to the sex toy debate reflects not merely moral condemnation but also the aversion to public display and support for sexual expressions that contest the established social order. We infer from the narratives and the analysis that the use of sex toys in the context of solo or partnered sex may not be the primary source of contention, but the public clamor for its decriminalization and the fact that a woman was making this call is what appears to invite greater scrutiny. This stems from perceptions regarding marital fidelity, where the use of sex toys could be considered as undermining male sexual hegemony or dislocating masculinity. The sentiments did not differ from these notions, as many expressed fear, ridicule, or moral outrage at the thought of women seeking pleasure outside the direct involvement of men, reinforcing the belief that sexual fulfillment should remain tied to male authority and control. This reflects broader patriarchal concerns about men maintaining power within intimate relationships and preserving traditional gender hierarchies, which have been part of the major concerns of both feminists and other activist groups. While a section of Ghanaians expressed optimism in an attempt to challenge these notions and cultural scripting, the overwhelmingly negative public backlash reveals the powerful and resilient nature of structural constraints. However, the positive sentiments identified in this study indicate a gradual shift toward recognizing individuals’ rights to make autonomous decisions regarding their sexual well-being. These emerging perspectives, although limited, suggest the possibility of more inclusive discussions about sexuality issues in Ghana, and this requires both cultural and structural shifts through inclusive sexuality education that recognizes sexual pleasure as a right, thereby challenging the conception that African societies limit women’s sexual options. Also, the findings point to the need for sexuality education and sexual and reproductive health policies to move beyond a primary focus on risk and reproduction and engage more directly with issues of sexual autonomy, pleasure, and bodily agency in culturally appropriate ways. Indeed, we agree with views such as those of Daniel Fiaveh, especially in his 2019 study on sex toys among women and other gender activists who suggest that women, like their male counterparts, exercise sexual choices in the “bedroom” so long as sexual pleasure is a “thing” they want to seek. So, so long as Madam Ursula Owusu, the Ghanaian women’s rights activist, is concerned, anti-LGBTQ legislation is not only targeted at gender and sexual minorities but also a threat to women’s sexual autonomy, and that should not be countenanced. We agree with Ursula’s feminist stance in that regard.

## Abbreviations

LGBTQ: lesbian, gay, bisexual, transgender, and queer

## References

[1] Agbeve AS, Fiaveh DY, Anto-Ocrah M (2022). Parent-adolescent sexuality communication in the African context: a scoping review of the literature. Sex Med Rev.

[2] Agbeve AS, Fiaveh DY, Anto-Ocrah M (2022). A Qualitative assessment of adolescent-parent sex talk in Ghana. Afr J Reprod Health.

[3] Anarfi JK, Owusu AY (2011). The making of a sexual being in Ghana: the state, religion and the influence of society as agents of sexual socialization. Sex Cult.

[4] Anarfi JK, Gyasi-Gyamerah AA (2014). Religiosity and attitudes toward homosexuality: views of Ghanaian university students. Res Soc Sci Study Relig.

[5] Okyerefo MPK, Fiaveh DY, Asante KT (2011). Religion as a tool in strengthening the democratic process in Ghana. J Afr Stud Dev.

[6] Amo-Adjei J (2024). Resistances to the implementation of comprehensive sexuality education curriculum in Ghana’s educational institutions. Int J Adolesc Youth.

[7] Fiaveh DY (2025). Sexuality, gender, and spiritual interventions: reflections on sexual world-making in Ghana. J Africana Relig.

[8] Asante GA, Mohammed WF, Appiah-Kubi AB (2024). The Oxford Handbook of Media and Social Justice.

[9] (2023). Catholic Bishops back anti-gay bill but want rights of homosexuals respected. Citi Newsroom.

[10] Fiaveh DY (2023). LGBQ+ in Ghana. Sociolinguist Stud.

[11] (2024). Using dildos and vibrators to please yourself, anal sex and other forms of unnatural carnal knowledge is still unlawful - Supreme Court. Graphic Online.

[12] (2023). Anti-LGBTQ bill: We shouldn’t criminalise sex toys – Ursula Owusu-Ekuful. MyJoyOnline.

[13] Döring N (2021). Encyclopedia of Sexuality and Gender.

[14] Suenzo FN, Pinch A, Cruz IF, Liang CA, Ross Arguedas A (2026). Knowledge and expertise in the digital age: how people engage with sex advice on TikTok. Int J Commun.

[15] Manduley AE, Mertens A, Plante I, Sultana A (2018). The role of social media in sex education: dispatches from queer, trans, and racialized communities. Fem Psychol.

[16] Foucault M (1978). The History of Sexuality.

[17] Butler J (2011). Gender Trouble: Feminism and the Subversion of Identity.

[18] Butler J (1993). Bodies That Matter: On the Discursive Limits of “sex".

[19] Nyanzi S (2014). Reclaiming Afrikan: Queer Perspectives on Sexual and Gender Identities.

[20] Tamale S (2014). Exploring the contours of African sexualities: religion, law and power. Afr Hum Rights Law J.

[21] Munnes S, Harsch C, Knobloch M, Vogel JS, Hipp L, Schilling E (2022). Examining sentiment in complex texts. A comparison of different computational approaches. Front Big Data.

[22] Nielbo KL, Karsdorp F, Wevers M (2024). Quantitative text analysis. Nat Rev Methods Primers.

[23] Bird S, Klein E, Loper E (2009). Natural Language Processing with Python.

[24] Medhat W, Hassan A, Korashy H (2014). Sentiment analysis algorithms and applications: a survey. Ain Shams Eng J.

[25] Eysenbach G, Till JE (2001). Ethical issues in qualitative research on internet communities. BMJ.

[26] Eysenbach G, Wyatt J (2002). Using the internet for surveys and health research. J Med Internet Res.

[27] Franzke AS, Bechmann A, Ess CM, Zimmer M (2020). Internet research: ethical guidelines 30. Association of Internet Researchers.

[28] Fiaveh DY (2019). Phallocentricism, female penile choices, and the use of sex toys in Ghana. Sexualities.

[29] Finnane M, Burchell G, Gordon C, Miller P (1992). The Foucault Effect: Studies in Governmentality with Two Lectures by and an Interview with Michel Foucault.

[30] Asante GA (2020). Anti-LGBT violence and the ambivalent (colonial) discourses of Ghanaian Pentecostalist-Charismatic church leaders. Howard J Commun.

[31] Otu KE (2021). Queer slacktivism as silent activism? the contested politics of queer subjectivities on GhanaWeb. Sexualities.

[32] Fiaveh DY (2020). Masculinity, male sexual virility, and use of aphrodisiacs in Ghana. J Mens Stud.

[33] Fiaveh DY, Okyerefo MP (2019). Femininity, sexual positions and choice. Sexualities.

[34] Fiaveh DY, Donkor M, Gooden A (2021). Gender and Sexuality in Ghanaian Societies.

[35] Dery I, Fiaveh DY, Apusigah AA (2019). “You Cannot Be Like That Here”: discourses of sexual identities among urban Ghanaian families. Gend Issues.

[36] Fiaveh DY, Mensah E (2023). Gender and sexuality in African discourses. Socioling Stud.

[37] Fiaveh DY (2024). Scoping review of discourses on masturbation in Africa: an Afrofeminist analysis. J Gend Stud.

[38] Signorini I (1973). Agɔnwole agyalɛ: the marriage between two persons of the same sex among the Nzema of southwestern Ghana. J Africanistes.

[39] Cinelli M, De Francisci Morales G, Galeazzi A, Quattrociocchi W, Starnini M (2021). The echo chamber effect on social media. Proc Natl Acad Sci U S A.

